# Health disparities and advertising content of women's magazines: a cross-sectional study

**DOI:** 10.1186/1471-2458-5-85

**Published:** 2005-08-18

**Authors:** Susan C Duerksen, Amy Mikail, Laura Tom, Annie Patton, Janina Lopez, Xavier Amador, Reynaldo Vargas, Maria Victorio, Brenda Kustin, Georgia Robins Sadler

**Affiliations:** 1 Rebecca and John Moores UCSD Cancer Center, University of California-San Diego School of Medicine, 9500 Gilman Drive, La Jolla, CA 92093-0658 USA; 2 Graduate School of Public Health, San Diego State University, San Diego, California, USA

## Abstract

**Background:**

Disparities in health status among ethnic groups favor the Caucasian population in the United States on almost all major indicators. Disparities in exposure to health-related mass media messages may be among the environmental factors contributing to the racial and ethnic imbalance in health outcomes. This study evaluated whether variations exist in health-related advertisements and health promotion cues among lay magazines catering to Hispanic, African American and Caucasian women.

**Methods:**

Relative and absolute assessments of all health-related advertising in 12 women's magazines over a three-month period were compared. The four highest circulating, general interest magazines oriented to Black women and to Hispanic women were compared to the four highest-circulating magazines aimed at a mainstream, predominantly White readership. Data were collected and analyzed in 2002 and 2003.

**Results:**

Compared to readers of mainstream magazines, readers of African American and Hispanic magazines were exposed to proportionally fewer health-promoting advertisements and more health-diminishing advertisements. Photographs of African American role models were more often used to advertise products with negative health impact than positive health impact, while the reverse was true of Caucasian role models in the mainstream magazines.

**Conclusion:**

To the extent that individual levels of health education and awareness can be influenced by advertising, variations in the quantity and content of health-related information among magazines read by different ethnic groups may contribute to racial disparities in health behaviors and health status.

## Background

Disparities in health status among racial and ethnic groups favor the Caucasian population in the United States on almost all major indicators [1-4]. African Americans have the highest annual death rates of any major racial or ethnic group from lung cancer, breast cancer, heart disease and stroke, as well as the highest incidence rates of AIDS and hypertension [5-9]. Hispanics have among the highest rates of hypertension, diabetes and AIDS, along with low rates of access to health care, diagnostic screenings and early prenatal care [1,8,10,11]. Both Blacks and Hispanics are more likely than non-Hispanic Whites to be obese and to report low levels of leisure time physical activity [5,8]. The observed disparities are only partially explained by differences in socioeconomic status [3,7,12].

Women, in particular, are generally less healthy and less likely to use health services if they belong to a minority group [13-16]. Black females born in the United States have a life expectancy 5.2 years shorter than that of their White counterparts [15]. African American women have elevated mortality rates from breast cancer, cardiovascular disease, and AIDS [5,7,16,17]. Both Black and Hispanic women have high rates of diabetes, diabetic complications and mortality, compared to Whites, and Mexican American women are particularly likely to have undiagnosed diabetes [10,13]. Relative to non-Hispanic Whites, Hispanic women also have high rates of cervical cancer, AIDS, teen pregnancy, and obesity, and are less likely to access basic health services [12,14,18,19].

The racial and ethnic imbalance in disease burden has been linked to a variety of factors such as income, education, health care access, cultural barriers, genetics, co-morbidities and environmental factors [15,20,21]. Among the many environmental factors may be a disparity in access to health-related information and ethnically aligned role models in the mass media [22]. Within the framework of the Health Belief Model and other cognitive theories of health behavior, knowledge is the essential underpinning of individual perceptions of health risks and the benefits of preventive action [23]. Repeated health-related messages in the mass media can contribute to individuals' knowledge and serve as cues, prompting individuals to take protective action [24-26]. Differential levels of awareness and knowledge about risks, symptoms and available treatments have been linked to ethnic and racial disparities in health status [13,27-32]. Manton et al. [33] noted in a report on racial health disparities that information shapes attitudes and "black attitudes toward disease, treatment, screening, and outcome may significantly affect their help-seeking behavior...." For instance, a disparity in access to basic information about breast cancer and mammography and in cues to screening action may contribute to the delayed detection of breast cancer in Black women compared to White women. That lag in detection is believed to be one reason Black women have a higher breast cancer death rate despite a lower incidence rate [16].

Social marketing theory suggests that the delivery of messages encouraging healthy behavior or other social change requires the same multi-pronged marketing methods employed in product sales [34-36]. While information alone is rarely sufficient to change behavior, it is a crucial element [24-28,30-37]. Beyond informing individuals, the mass media can play an important role in setting the public agenda, stimulating public attention to issues, and offering repeated cues to action [38,39]. Innovation diffusion theory, also known as technology transfer, holds that the mass media can be pivotal in influencing beliefs and attitudes that lead to behavior changes [37,40].

Marketing messages often are targeted to specific population segments, presumably for the purpose of increasing the company's market share among that segment of the population. Garfield reported that advertising for alcoholic beverages focused on magazines with high adolescent readership [41]. Smith and Malone explored industry's decision to focus on marketing to the gay and lesbian community as a strategy for bolstering market share [42]. Balbach et al. showed the importance of strategically placed advertising designed to secure an increased share of the African American market when they examined the marketing and advertising strategies associated with the Uptown brand of the R.J. Reynolds Tobacco Company [43].

While women access health information from a variety of sources, multiple surveys have shown lay magazines to be one key source of health information for women [44-46]. A study by Sadler et. al found that more African American women listed mass media as a key source of information on breast cancer and diabetes than listed physicians, and print media were listed three times as often as electronic media [47]. In a national Gallup poll in September 2002, 51 percent of Americans – and 56 percent of those aged 50 and above – said they got a great deal or moderate amount of health and medical information from magazines [48].

Although many Black and Hispanic women read mainstream White-oriented magazines [49], it is likely that readers give more serious consideration to, and are more influenced by, health-related content in magazines that present them with culturally aligned role models and information of personal relevance [50-52]. The few studies that have examined the health-related content of magazines by the magazines' ethnic orientations have found some significant differences in the amount of health information directed at Black female and White female audiences. Gerlach et al. found that coverage of colon cancer was greatly underrepresented in both White and Black women's magazines, compared to the risk the disease poses to women, but African American magazines contained many fewer articles on colon cancer than did White-oriented magazines [53]. Omonuwa reported more than four times as many advertisements for prescription and nonprescription drugs in White women's magazines as in those directed at Black women, without adjusting for the number of pages in the magazines [54]. In a six-year study of nutritional advertising in three magazine, Pratt and Pratt found what they called a "deluge" of alcohol advertisements in two major magazines for Black women: 62% of the food and beverage ads in *Ebony *and 47% in *Essence *were for alcohol [22]. By contrast, the White-oriented *Ladies' Home Journal *had only 2% alcoholic beverage ads.

This study reports on the development and testing of a methodology designed to facilitate a more in-depth qualitative and quantitative content analysis of the health information and cues provided in lay magazines [55]. For this first assessment, the researchers focused on an analysis of the advertising content of women's magazines.

## Methods

The authors tested the hypothetical statement: Magazines oriented primarily toward Caucasian women contain significantly more health-promoting and less health-diminishing advertising content than those targeting African American and Hispanic women.

To test this hypothesis for health-related advertisements, assessments were made of advertising content in the four highest circulating, general interest magazines aimed primarily at Black women and the four comparable magazines aimed at Hispanic women, compared to advertising content in the four highest-circulating magazines aimed at a predominantly White readership. General interest magazines were defined as those covering a wide range of topics of interest to women; magazines more narrowly focused on fashion, beauty and relationships, such as *Cosmopolitan*, were excluded. Circulation and orientation were assessed using the SRDS Consumer Magazine Advertising Source. *O, The Oprah Magazine*, although launched by an African American celebrity, was not included among the African American magazines because the majority of the readership was White and a content review of role models pictured in the magazine revealed a mainstream rather than an African American orientation. Nor was the magazine's circulation high enough to be included among the mainstream magazines analyzed. Table 1 displays the characteristics of the magazines for which comparable information was available. While health disparities affecting the Asian-Pacific Islander community are of concern, comparable women's journals for that population were not available to be included in this analysis. This is presumed to be at least partly because the Pan Asian community is composed of many smaller ethnic groups, each with its own unique language, culture, and print media.

**Table 1:** Magazine readership demographics

**Magazines**	**Paid Circulation**^a^	**Issues/Year**^a^	**Aver. Pages/Issue**	**% Female**^c^	**% White**^b^	**% Black**^b^	**Median Age**^c^	**Median Household Income**^c^
***Mainstream***								
Family Circle	4,712,548	15	173	90	89	9	48.2	$49,221
Good Housekeeping	4,527,447	12	201	87	87	11	47.3	$52,609
Ladies' Home Journal	4,100,675	12	159	94	90	8	48.0	$52, 634
Woman's Day	4,257,742	17	155	94	87	12	47.7	$50,356
***African American***								
Ebony	1,785,139	12	182	61	7	93	39.4	$38,678
Essence	1,053,484	12	171	75	6	92	35.8	$40,698
Heart and Soul	308,899	10	104	85	NA	NA	37.0	$47,082
Upscale	224,649	9	124	70	NA	NA	31.0	$72,500
***Hispanic American***								
Latina (English)	230,503	12	115	94	NA	NA	31.0	$45,807
Latina Style (English)	152,561	5	62	NA	NA	NA	30.0	$73,000
Cristina (Spanish)	102,194	12	103	NA	NA	NA	18–49	37% over $35,000
Vanidades (Spanish)	90,598	26	132	NA	NA	NA	25–54	54% over $35,000

^a ^Sources: SRDS Consumer Magazine Advertising Source, May 2002 and Audit Bureau of Circulations, December 2001

^b ^Source: Mediamark Fall 2000. White and Black categories include Hispanic.

^c ^Source: magazine publishers

The sample included all issues of the 12 selected magazines published in June, July, and August 2002. During this time frame, holiday specific advertising would have been focused on American holidays (e.g. the Fourth of July) rather than culturally specific holidays like Kwanzaa or Cinco de Mayo. Publication frequency varied from bi-monthly to bi-weekly, resulting in a total of 12 issues of mainstream magazines, 10 African American, and 14 Hispanic American during the three months. These differences in publication frequency, as well as differences in the magazines' page sizes and total number of pages, were accounted for by assessing each of the magazines' total square inches of printed pages during the three-month assessment period. This allowed the investigators to make a proportional assessment, as well as a "dose intensity" assessment of the potentially healthy and potentially unhealthy advertising impact on each of the three ethnic groups. Comparisons were then made between the absolute and relative amounts of health-related ads in the mainstream magazines and in those aimed at African Americans and Hispanics.

Data were collected and analysis completed in 2002 and 2003. Abstracting guides were developed to code the size and page numbers of all advertisements and to code the advertising content into 48 categories. (Classified ads were not individually analyzed because of their small size and hence limited impact.) Of the 48 content categories, 35 were considered health-related because the advertised products had the potential to affect health. The remaining 13 were broad categories such as entertainment, retail, beauty supplies, and financial services. The 35 health-related categories included:

• 16 advertising categories related to health promoting medical treatments or products judged to have a potentially positive impact on health (i.e., drugs, vitamins, exercise equipment, sunscreen, clinical trials participation, and prevention of smoking or substance abuse). For this analysis, prescription drug ads were considered positive because they provided general health information or at least awareness about related health conditions, not because of the drugs themselves.

• 6 advertising categories promoting products with potentially negative health consequences (i.e., cigarettes, alcohol, and medical treatments with no apparent value).

• 13 food and non-alcoholic beverage advertising categories, including:

     5 with potentially positive or healthy impact;

     4 with potentially negative or unhealthy impact;

     4 with potentially mixed health impact.

Food and beverage ads were designated as having potentially healthy or unhealthy impact based on subjective evaluations of the usual nutritive value, fat content and energy density of the primary product advertised. Therefore, all advertisements for vegetables, fruit, 100% fruit juice, milk, soymilk, yogurt, water, tea, plain coffee, plain nuts, veggieburgers, baked beans, plain bread, plain rice and non-chocolate granola bars were coded as healthy. All foods and vegetables advertised as weight-loss, low-fat or reduced-calorie, including diet sodas, also were coded as healthy. Foods and beverages coded as unhealthy included: candy, ice cream, gelatin desserts, other desserts, chips and other high-fat/low-nutrient snacks (e.g. cheese puffs), granola bars containing chocolate, fast food, non-diet sodas, juice drinks with added sugar and coffee drinks with added sugar and creamer. To avoid subjective decisions in coding, all fast food restaurant advertisements were considered unhealthy, even if relatively healthy menu items were highlighted. The advertisements were considered promotions of fast food chains, where most food and drink products sold are high-fat or high-sugar.

Some types of foods were coded as having mixed health impact because they had both healthy and unhealthy aspects (e.g., foods with high energy density but also substantial nutrient value). These included red meat/processed meat products such as hot dogs, cheeses, all cereals except plain rolled oats, all condiments not advertised as low-fat or low-calorie, macaroni and cheese mixes, and all refrigerated or frozen packaged meals that were not advertised as weight-loss or low-fat products. All condiments and cereals were assigned the neutral category of "mixed" to avoid the need for subjective distinctions by the reviewers and to simplify the process. The majority of advertisements in those categories were for relatively high-fat or high-sugar items.

Coding was also developed to quantify the photographic racial and ethnic role models presented in advertisements as White, Black, Hispanic, and Other. The standard used for counting people in the photos was to include everyone whose face was at least half visible but not photos taken from the back or of body parts other than faces. Photos of all sizes were counted equally, but the number of advertising pages with any faces also was reported to partially control for the differential impact of very differently sized faces. In the very rare cases in which there was doubt about whether a face was White or Black, the photo was coded as "other" and was excluded from analysis. In the magazines reviewed, there were almost no faces coded as "other," which included Pan Asian. Since distinguishing Hispanic faces with even a modest degree of confidence proved difficult, even for the four Hispanic research team members, the attempt to count Hispanic faces was abandoned. (Coincidentally, one of the magazines studied contained an article about the wide racial variation among Hispanics: "Will the real Latina please stand up?" *Latina*, July 2002, page 80).

Two reviewers independently analyzed each of the 36 magazines under study, with the pairs selected randomly from among eight researchers. The two magazines published in Spanish were reviewed by varying pairs of the five researchers literate in Spanish.

To confirm the consistent application of the content analysis guide, pilot testing and honing of the guide was done to establish a baseline inter-rater reliability rate of 95% (kappa = 0.92) and maintenance of this rate was reconfirmed throughout the data collection process. A third reviewer rechecked the coding of both primary reviewers.

When the first two reviewers disagreed and the third was unable to reach a decision, the coding was discussed at a meeting of all reviewers and senior authors, and a consensus decision was reached in all cases. In the absence of recommended guidelines for health-related advertising content, the advertising content and quantity in the mainstream magazines were used as the points of reference to which the African American and Hispanic magazines were compared.

## Results

Table 2 displays the cumulative advertising space for each of the three clusters of ethnic magazines for the three-month period under investigation. After taking the number of pages, page sizes, and publication frequency into account, the proportion of magazine space devoted to all advertising was similar for the African American and mainstream magazines (approximately 50%), but lower for the Hispanic magazines (30%). Approximately half of all advertisements in mainstream magazines were health-related, more than double the proportion in the Black and Hispanic magazines.

**Table 2:** Space devoted to health-related advertising

***Square Inches***				Health-Related Ads		
				
Magazine Type	Total Sq. Inches over 3 months^a^	Advertising as % of Magazine Space	Health-Related Ads as % of All Ad Space	% Positive	% Negative	% Mixed
Mainstream	171,671	49.9	51.9	65.0	17.1	18.0
African American	130,666	47.3	22.4	58.0	28.7	13.3
Hispanic American	135,456	29.5	25.9	48.9	43.7	7.5
						
***Number of Ads***				All Health-Related Ads		
				
Magazine Type	Number of Pages over 3 months	Number of Ads^b^	Health-Related Ads as % (N) of all Ads	% Positive	% Negative	% Mixed

Mainstream	2,066	943	50.0 (470)	61.5	17.4	21.1
African American	1,518	684	20.5 (140)	52.9	30.0	17.1
Hispanic American	1,570	466	23.6 (110)	52.7	39.1	8.2

^a ^Magazine pages average 84 square inches

^b ^Some ads are more than one page

In terms of the proportion of both square inches of advertising space and the actual number of advertisements, the African American and Hispanic magazines contained fewer advertisements with potentially healthy impact and more with potentially unhealthy impact than the mainstream magazines (Table 2). Table 3 lists the types of advertisements with positive, negative, and mixed impact, clustered by the magazines' ethnic orientation.

**Table 3:** Number of health-related ads by topic

	**Mainstream N**	**African American N**	**Hispanic American N**
***Health Related Ads (N=)***	470	140	110
**Positive Health Related Ads**			
Prescription Drugs	65	20	1
Over the Counter Drugs, Vitamins, and Supplies^a^	121	25	22
Medical Treatments and Procedures	2	4	2
Smoking and Drug Abuse Prevention	18	4	6
Sun Protection	5	1	0
Exercise Equipment and Facilities	1	0	1
Healthy Foods and Beverages	47	14	12
Weight Loss and Low Calorie Foods and Beverages	20	3	3
Health-Related Organizations and Charities	10	3	11
Clinical Trials Participation Promotion	0	0	0
**Subtotal Positive Ads**	**289**	**74**	**58**
**Negative Health Related Ads**			
Cigarettes	15	11	12
Alcohol	0	12	3
Unhealthy Foods and Beverages	64	15	25
Fast Food Restaurants	3	4	1
Pseudo-Treatments^b^	0	0	2
**Subtotal Negative Ads**	**82**	**42**	**43**
**Health Related Ads of Mixed Impact**			
Meats	14	4	5
Condiments	53	7	2
Cereals	11	2	0
Miscellaneous and Packaged Meals	21	11	2
**Subtotal Mixed Ads**	**99**	**24**	**9**

^a ^Includes dietary supplements, vision and hearing products, vitamins, medical supplies, and drugs purchased without a prescription

^b ^Ads for questionable "blood treatments"

The mainstream magazines contained 28% of all cigarette and alcohol ads published in the 12 magazines during the three-month study period, while they had almost two-thirds of all preventive advertisements related to smoking cessation and alcohol and drug abuse prevention (Table 3). Similar to the earlier findings by Pratt and Pratt [22], there were no alcohol advertisements in the White-oriented magazines, 12 in the African American magazines and 3 in the Hispanic magazines. Advertisements for over-the-counter drugs, vitamins, and medical supplies were placed in mainstream magazines most frequently; 72% of 168 such ads were in the mainstream magazines, 15% in African American magazines and 13% in Hispanic magazines. Except for alcohol and fast food, mainstream magazines had a higher percentage of all categories of food and beverage advertisements than either of the other two ethnic groups of magazines. In all three types of magazines, 28–30% of the food and beverage ads were for healthy and low-calorie products, but overtly unhealthy food and drink ads (other than alcohol) comprised 52% of food and beverage advertisements in Hispanic magazines, compared to 32% in Black magazines and 29% in mainstream magazines.

Of the 86 prescription drug ads in the 12 magazines during the three-month study period, 76% were placed in the four mainstream magazines. Only one prescription drug ad appeared in any of the Hispanic magazines during the three months studied. Prescription drugs and cigarettes were the products with the most multi-page ads. Table 4 displays the health conditions for which prescription drugs were advertised, illustrating a disparity in the placement of prescription drug advertising. Although hypertension disproportionately affects African Americans, ads for hypertension drugs were confined to mainstream magazines, as were most of the allergy, diabetes, arthritis and cholesterol drug ads. However, ads for birth control, HIV medications and Viagra appeared only in the African American magazines. Advertisements to promote clinical trials participation were completely absent from all magazines.

**Table 4:** Prescription drug ads by category

**Drug Category**	**Mainstream**	**African American**	**Hispanic American**
Allergy	13	2	1
Diabetes	11	6	
Heartburn/reflux	7		
Arthritis	6		
Chemo-related Anemia	5		
Cholesterol	4	1	
Hypertension	4		
Acne	3		
Hormone Replacement Therapy	3		
Nail Fungus	3		
Yeast Infection	3	4	
Osteoporosis	2		
Alzheimer's	1		
Birth Control		3	
HIV		3	
Erectile Dysfunction (Viagra)		1	
**TOTAL**	**65**	**20**	**1**

Table 5 displays the analysis of the identifiable White and Black faces. Those advertisements selling products with negative health impact in the African American magazines frequently (55%) featured faces of African Americans. In contrast, comparable health-diminishing ads in the mainstream magazines rarely (6%) included any people at all, although 58% of the health-promoting ads in mainstream magazines featured White faces.

**Table 5:** Racial/ethnic representation on health-related ad pages (by face count in photographs)

	**Mainstream**	**African American**
**Positive Health-Related Ads**	Number of ad pages = 238*	Number of ad pages = 73*
#Ad Pages with White Faces	**138 (58%)**	8
#Ad Pages with Black Faces	28	**30 (41%)**
Total # of White Faces	**232**	15
Total # of Black Faces	43	**47**
**Negative Health-Related Ads**	Number of ad pages = 83*	Number of ad pages = 42*
#Ad Pages with White Faces	**5 (6%)**	4
#Ad Pages with Black Faces	1	**23 (55%)**
Total # of White Faces	**8**	9
Total # of Black Faces	3	**47**

* The total number of ad pages includes pages with no faces or indistinguishable faces

Analysis of the specific page locations of different types of ads within the magazines yielded cells that were too small to draw conclusions with only the three-month period of evaluation used for this study. Further evaluation of this outcome measure is warranted in a larger study.

## Discussion

While many factors that contribute to health disparities are extremely difficult to change, lifestyle choices can be influenced by selective advertising, marketing, and public relations. In addition to selling health-related products, advertisements can contribute to public awareness and access to information about health conditions. This can contribute to readers' perceptions of personal risk status, knowledge of how to lower risks and ability to weigh the pros and cons of selecting healthy alternatives. These are all key elements of the Health Belief Model and other theoretical frameworks. Indeed, that informative potential is one of the primary rationales for allowing direct-to-consumer advertising of prescription drugs [56].

To the extent that individual levels of health education and awareness can be influenced by advertising, variations in the quantity, quality, and content of health-related information among magazines read by different ethnic groups may contribute to disparities in health behaviors and health status. This study compared the dose intensity of health-promoting and health-diminishing magazine advertisements in African American, Hispanic and mainstream magazines. The shibboleth of marketing is that multiple messages must be received to achieve top-of-mind awareness regarding a specific message. From this perspective, the actual number of messages delivered to each cultural group is as important as the proportion of messages compared to other types of messages. In addition to the "dose intensity" of messages, the Health Belief Model suggests that the narrowness of the time frame in which social marketing messages are delivered and the alignment of the message with the intended recipient's culture should influence the intensity of the message.

In comparison to mainstream magazines, magazines specifically created for African American and Hispanic American women were smaller, less frequently published (with the exception of *Vanidades*), and had proportionally more potentially harmful health-related advertising and less potentially health-promoting advertising. None of the magazines in the study had any advertisements recruiting women for clinical trials or supporting the idea of participation in health-related research, indicating a missed opportunity to address a critical issue in women's health, and especially minority women's health. The limited pharmaceutical advertising in minority women's magazines further reduced those readers' exposure to information related to the benefits of clinical trials.

The finding that Black faces are more frequently used to advertise products with negative health impact than are White faces deserves further research. In the African American magazines, a higher proportion of negative health-related ads than positive ads contained Black faces. In the mainstream magazines, however, White faces were almost completely absent from negative health-related ads and appeared frequently in positive health-related ads. The possible reasons for this advertising discrepancy are beyond the scope of this paper, but the ramifications of the disproportionate exposure to negative role models are potentially significant.

Generalizations from this study must be drawn with caution. While this pilot study evaluated the advertising content of lay magazines with national readership, it included only a three-month period of publication. Some of the magazines studied did not precisely fit the inclusion criterion of "general women's magazines." *Ebony *is not officially a women's magazine, but is "a Black-oriented, general, picture magazine dealing primarily with contemporary topics." It was included because the magazine regularly contains articles on fashion, beauty, recipes, relationships, tips for mothers and other features typical of women's magazines, and 61% of its readers are women [49,57].

In addition, since journals focused on Pan Asian women were not included in this analysis, generalizations cannot be extended to this community. Inclusion of Pan-Asian journals might have helped distinguish between socio-economic factors and ethnicity since Pan Asian families, on average, have a higher household income than any other minority group [58]. However, data on median household income of magazine readers in this study (Table 1) do not suggest widely disparate socio-economic status between the mainstream, African American, and Hispanic American readers.

Another limitation of this study is that age disparities existed among readers of the highest-circulation women's magazines in the three racial/ethnic categories. The median age of readers of the mainstream magazines was about 10 years higher than that of the African American magazines, as reported by the publishers. Median ages were not available for two of the Hispanic magazines, but the age ranges provided indicate that the median was probably similar or slightly lower than that of the African American readers. This is a potential confounder that warrants further investigation.

One of the goals of this study was to test and refine our data collection instruments and coding guidelines. Categorizing food advertisements as having positive or negative health impact was problematic because the health value of many foods depends on the context and manner in which they are eaten. While this is especially true for the food and beverage items classified as mixed, it also applies to all other foods. Milk, for instance, was classified as a healthy beverage although drinking large quantities of whole milk can contribute to high cholesterol and obesity. A more precise system might evaluate the health impact of each ad, so that, for example, a cereal advertisement focusing on a recipe for making a high-fat dessert with the cereal would be classified as negative.

## Conclusion

These findings suggest that health information disparities in the advertising content of lay magazines could contribute to health disparities among women of differing ethnic communities. Since women often make health-related and purchasing decisions that affect their family members, disparities in women's health knowledge, attitudes, and behaviors can have implications for entire families.

## Competing interests

The author(s) declare that they have no competing interests.

## Authors' contributions

SD and AM were involved with study design, data collection, data analysis, and manuscript preparation. LT, AP, JL, XA, RV, and MV participated in data collection and manuscript preparation. BK oversaw the integrity of the database and assisted with mathematical calculations within the database. GS conceived of the study, secured the research funding, participated in study design, coordination, and manuscript preparation.

## Pre-publication history

The pre-publication history for this paper can be accessed here:


